# Supplementary material for: Echocardiographic Assessment of Patients with Glycogen Storage Disease in a Single Center
Source: Int J Environ Res Public Health. 2023 Jan 25;20(3):2191. doi: 10.3390/ijerph20032191 (PMC9916218; doi:10.3390/ijerph20032191)
Supplement: Supplementary file 1 [file ijerph-20-02191-s001.zip › ijerph-2121419-supplementary.pdf]

Supplementary Table S1. Correlation analysis between the patients' characteristics and cardiac function.

**Variables**		**Age**	**BMI z score**	**LV mass z score**	**Uric acid**	**lactate**	**Total col**	**TG**	**AST**	**ALT**	**CK**
EF	r	-0.175	0.065	0.221	-0.116	-0.105	0.085	0.163	0.394*	0.471*	0.284*
	P value	0.206	0.643	0.109	0.404	0.450	0.543	0.238	0.003	0.002	0.038
FS	r	-0.138	0.072	0.243	-0.088	-0.080	0.120	0.181	0.441*	0.445*	0.265*
	P value	0.321	0.610	0.077	0.526	0.563	0.385	0.189	0.002	<0.001	0.053
**E/A (z)**	r	-0.171	**-0.286***	-0.162	-0.153	-0.081	-0.149	-0.246	-0116	-0.131	-0.142
	P value	0.217	**0.038**	0.242	0.271	0.560	0.283	0.073	0.403	0.346	0.306
**E/e' (z)**	r	0.249	0.226	0.109	0.214	0.237	0.106	**0.286***	0.013	0.074	-0.119
	P value	0.072	0.108	0.439	0.124	0.087	0.451	**0.038**	0.925	0.599	0.395
RV S'(z)	r	0.049	0.107	0.092	0.222	0.094	0.181	0.225	0.116	0.166	0.049
	P value	0.727	0.447	0.510	0.107	0.499	0.190	0.102	0.404	0.231	0.725
Strain (z)	r	-0.208	0.032	-0.279*	-0.217	-0.212	-0.224	-0.114	0.051	0.063	-0.032
	P value	0.132	0.821	0.041	0.116	0.123	0.103	0.410	0.715	0.652	0.818

AST, aspartate transaminase; ALT, alanine transaminase; BMI, body mass index; CK, creatinkinase; EF, ejection fractioin; FS, fractional shortening; LV, left ventricle; LVPWd, left ventricular posterior wall thickness diameter; IVSd, interventricular septum diameter; TG, triglyceride; Total Col., total cholesterol; z, z-score
